# Continuous hydrogenation of ethyl levulinate to γ-valerolactone and 2-methyl tetrahydrofuran over alumina doped Cu/SiO_2_ catalyst: the potential of commercialization

**DOI:** 10.1038/srep28898

**Published:** 2016-07-05

**Authors:** Junlin Zheng, Junhua Zhu, Xuan Xu, Wanmin Wang, Jiwen Li, Yan Zhao, Kangjian Tang, Qi Song, Xiaolan Qi, Dejin Kong, Yi Tang

**Affiliations:** 1 Shanghai Research Institute of Petrochemical Technology SINOPEC, Shanghai 201208, China; 2 Department of Chemistry, Fudan University, Shanghai, 200233, China

## Abstract

Hydrogenation of levulinic acid (LA) and its esters to produce γ-valerolactone (GVL) and 2-methyl tetrahydrofuran (2-MTHF) is a key step for the utilization of cellulose derived LA. Aiming to develop a commercially feasible base metal catalyst for the production of GVL from LA, with satisfactory activity, selectivity, and stability, Al_2_O_3_ doped Cu/SiO_2_ and Cu/SiO_2_ catalysts were fabricated by co-precipitation routes in parallel. The diverse physio-chemical properties of these two catalysts were characterized by XRD, TEM, dissociative N_2_O chemisorptions, and Py-IR methods. The catalytic properties of these two catalysts were systematically assessed in the continuous hydrogenation of ethyl levulinate (EL) in a fixed-bed reactor. The effect of acidic property of the SiO_2_ substrate on the catalytic properties was investigated. To justify the potential of its commercialization, significant attention was paid on the initial activity, proper operation window, by-products control, selectivity, and stability of the catalyst. The effect of reaction conditions, such as temperature and pressure, on the performance of the catalyst was also thoroughly studied. The development of alumina doped Cu/SiO_2_ catalyst strengthened the value-chain from cellulose to industrially important chemicals via LA and GVL.

The catalytic conversion of biomass to fuels and value-added chemicals has been the subject of intense research efforts during the past two decades. Especially, lignocellulosic materials have been targeted as promising feedstock in that this kind of biomass does not compete with feed or food production. Raw lignocellulosic biomass is primarily composed of the biopolymers of cellulose, hemicellulose, and lignin. Levulinic acid (LA) can be commercially produced by acid catalyzed hydrolysis of biomass-derived cellulosic carbohydrates at low cost^1^,^2^,^3^. National Renewable Energy Laboratory (NREL, USA) identifies LA as one of top 12 platform chemicals that can be produced from biomass in their biomass program^4^. γ-valerolactone (GVL) is an important platform molecule and has the potential to be used in many areas including solvent^5^,^6^,^7^, biofuels production^8^,^9^,^10^,^11^, intermediate in the fine chemicals synthesis^12^,^13^,^14^, and so on. Because 2-methyl tetrahydrofuran (2-MTHF) is miscible with gasoline at all proportions and hydrophobic, it is projected to be used as a transportation fuel extender^1^. 2-MTHF has been successfully road-tested in fuel blends. It is a component of P-series fuels that are recently classified as alternative fuels by the US Department of Energy^15^. Due to both the attainability of LA as a feedstock and the potential usability of GVL and 2-MTHF, the targeted production of GVL and 2-MTHF from LA has been the focus of considerable academic and industrial research efforts^1^.

Generally, GVL and 2-MTHF can be produced through selective hydrogenation of biomass-derived LA and levulinate esters on either homogeneous or heterogeneous catalysts^16^,^17^,^18^,^19^. Homogeneous catalyst systems obviously have serious drawbacks of catalyst synthesis, recovery, and recycling for commercial application. The high boiling point of GVL (207–208 °C) makes product/catalyst separation by means of distillation uneconomical. The heterogeneous hydrogenation process is preferable considering the feature of high efficiency. A substantial number of heterogeneous catalysts have been studied in this reaction, with the most common being supported hydrogenation metals. Most of the heterogeneous catalysts are based on noble metals like ruthenium^20^, palladium^21^, and gold^22^. However, heterogeneous catalysts based on noble metals have obvious disadvantages, such as high cost, hard manufacture, and vulnerability to poison. Thus, base metal catalysts with high activity and selectivity are highly desired for the purpose of industrial application.

Copper loaded heterogeneous catalysts are good candidates for the continuous reduction of LA and its ester EL to GVL and 2-MTHF. One early method considered for the commercial-scale manufacturing of GVL is vapor-phase continuous LA hydrogenation developed by the Quaker Oats Company in the 1950s, in which the catalyst is prepared from CuO and Cr_2_O_3_^23^. However, the usage of chromium promoter is harmful to the environment and human health. Copper supported on γ-Al_2_O_3_ catalyst is fabricated for the vapor-phase hydrogenation of LA to GVL^18^. Unacceptably, it is observed that the conversion is just 95% in the initial period and further decreases to 80% at 25 h of reaction time. The selectivity of valeric acid at 250 °C is as high as 7% when the conversion of LA was just 72%, which results in severe leaching of copper. Cu-ZrO_2_ nanocomposite catalyst for the batch hydrogenation of LA and its ester to GVL is also investigated^24^. The selectivity of GVL decreases gradually with more catalyst recycles when the solvent is water. The leaching of copper is a problem, and the formation of a copper-carboxylate complex accounts for this effect. It is also reported that LA can be hydrocyclized to GVL and 2-MTHF over nanocomposite copper/SiO_2_ catalysts^25^. But the reaction temperature is as high as 265 °C. For the sake of catalyst sintering as well as partial oxidation of Cu^0^ state, copper-based catalysts are generally not considered to be effective hydrogenation catalyst at high reaction temperatures like above 250 °C^26^,^27^. It is found that the adopted reaction temperatures are all above 200 °C after comprehensive survey on the previous literatures. And furthermore, substantial by-products form, such as 1-pentanol, 2-pentanol, valeric acid, 1,4-pentanediol, and angelica lactone, which deteriorates the overall selectivity of targeted products. Based on the information provided in the previous literatures, there are four main problems to be solved before this kind of catalyst can be successfully put into commercial use, i.e., utilization of hazardous promoters, poor stability because of leaching of copper by the attack of LA substrate and valeric acid by-product, too high reaction temperatures because of low catalytic activity, and low selectivity of target products.

Aiming to develop a commercially feasible base metal catalyst for the production of GVL from LA, with satisfactory activity, selectivity, and stability, an Al_2_O_3_ doped Cu/SiO_2_ catalyst is successfully developed and systematically assessed in the continuous hydrogenation of ethyl levulinate (EL) in a fixed-bed reactor. In comparison, a Cu/SiO_2_ catalyst is also produced and tested for the continuous hydrogenation of EL. The effect of acidic property of the SiO_2_ substrate on the catalytic properties is investigated. To justify the potential of its commercialization, significant attention is paid on the initial activity, proper operation window, by-products control, selectivity, and stability of the catalyst. The effect of reaction conditions, such as temperature and pressure, on the performance of the catalyst is also thoroughly studied. The influence of alumina doping in the SiO_2_ support is manifested based on comprehensive characterizations and catalytic performance test.

## Catalysts characterization

**Figure 1:**
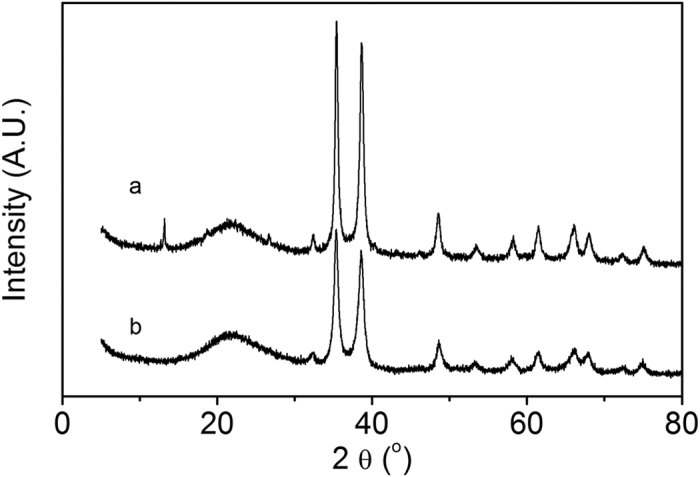
XRD patterns of the two catalysts: (**a**) CuO/SiO_2_; (**b**) CuO/Al_2_O_3_-SiO_2_.

**Figure 2:**
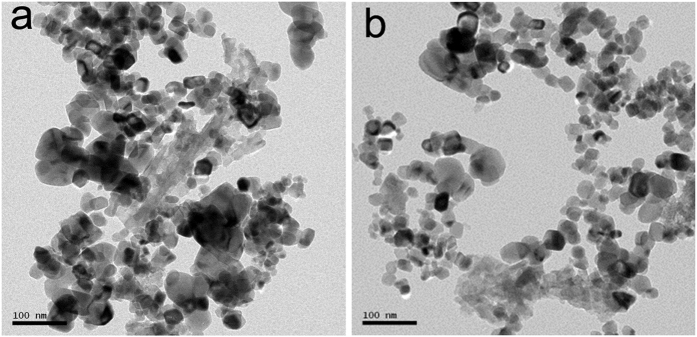
TEM images of catalysts: (**a**) CuO/SiO_2_; (**b**) CuO/Al_2_O_3_-SiO_2_.

The XRD patterns of CuO/SiO_2_ and CuO/Al_2_O_3_-SiO_2_ are shown in Fig. 1. The diffraction peaks at 35.42° and 38.65° are characteristic of CuO phase. Besides, for CuO/SiO_2_, the diffraction peaks of CuO are sharper than that of CuO/Al_2_O_3_-SiO_2_, indicating that the CuO particle sizes of CuO/Al_2_O_3_-SiO_2_ are smaller. TEM micrographs of two catalysts are displayed in Fig. 2. In CuO/SiO_2_ catalyst, the CuO particles are around 90 nm in diameter. In CuO/Al_2_O_3_-SiO_2_ catalyst, the CuO particles distribute around 20–50 nm. The CuO/SiO_2_ catalyst exhibits larger CuO particle size than the alumina doped Cu/SiO_2_ catalyst, which agrees with the information provided by XRD patterns. Table 1 gives the Cu dispersion data of these two catalysts as determined by dissociative N_2_O chemisorptions on surface copper atoms. The reduced Cu particles of Cu/Al_2_O_3_-SiO_2_ are averagely 23.02 nm in diameter, while those of Cu/SiO_2_ are 27.01 nm in diameter. The Cu particle size in Cu/Al_2_O_3_-SiO_2_ catalyst is also a bit smaller than that of its counterpart. Calculated from the XRD patterns of H_2_ reduced catalysts (as shown in Figure S1), the copper particle sizes of Cu/SiO_2_ and Cu/Al_2_O_3_-SiO_2_ are 22.40 nm and 19.10 nm, respectively, which agrees with the dispersion data obtained by dissociative N_2_O chemisorptions method.

To further elucidate the differences between two catalysts, the acidic properties of them are studied by Py-IR spectroscopy as displayed in Figure S2 and Table 2. Normally, the peak at 1450 cm^−1^ and 1540 cm^−1^ are ascribed to Lewis acidic sites and Brönsted acidic sites, respectively. The Cu/SiO_2_ catalyst only has very weak Lewis acidic sites, as indicated by the desorption signal at 200 °C and 250 °C. Whereas, the Cu/Al_2_O_3_-SiO_2_ catalyst possesses stronger Lewis acidic sites, as indicated by the stronger desorption signals at 200 °C and 250 °C, as well as the additional desorption signals at 300 °C (Figure S2). Considering the procedure difference of catalyst fabrication, the doping of Al_2_O_3_ may help to the better dispersion of CuO particles. Yuan *et al*. also reported better dispersion of Cu in boric acid doped Cu-SiO_2_ catalyst for vapor-phase hydrogenation of dimethyl oxalate when the boron doping was in the range of 0.51 wt%^28^.

### Catalytic performances of Cu/SiO_2_ and Cu/Al_2_O_3_-SiO_2_ catalysts

**Figure 3:**
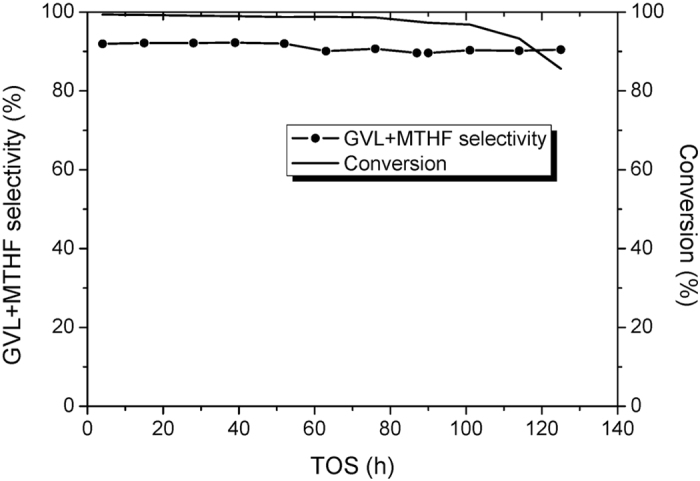
Catalytic performance of the Cu/SiO_2_ catalyst.

The Cu/SiO_2_ catalyst is tested for the hydrogenation of EL in a fixed bed reactor. The activities and products selectivities under different temperatures are also listed in Table 3. Below 200 °C, the EL feedstock can not be completely converted. The catalytic stability of this catalyst is displayed in Fig. 3. In the first 100 h, EL is almost entirely converted under 230 °C. However, the Cu/SiO_2_ catalyst deactivates drastically after 100 h time-on-stream. Therefore, the stability of the Cu/SiO_2_ catalyst can not satisfy the requirement of long-term operation. The total selectivity of GVL and 2-MTHF remains at around 90%. The distribution of the all the products at 54 h was thoroughly analyzed. As listed in Table 3, the selectivities of 2-MTHF, GVL, 2-pentanol, 1-pentanol, 1,4-pentanediol, and ethyl valerate are 7.18%, 85.06%, 2.95%, 1.24%, 2.04%, and 0.10%, respectively, besides 1.43% unidentified products.

**Figure 4:**
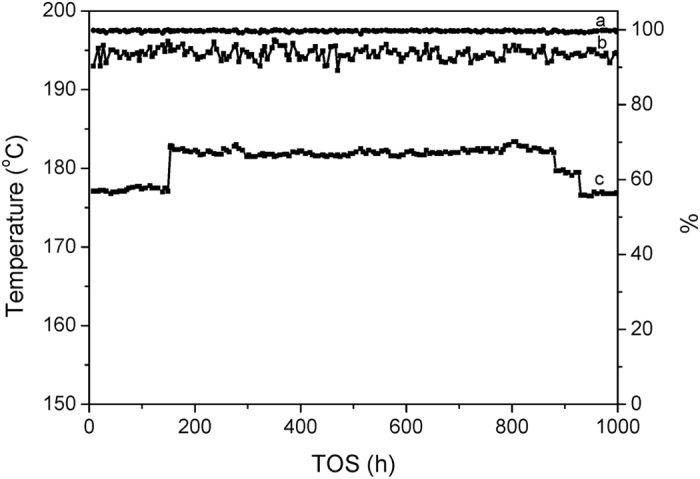
1000 h continuous assessment of the Cu/Al_2_O_3_-SiO_2_ catalyst for EL hydrogenation: (**a**) EL conversion, %; (**b**) GVL and 2-MTHF selectivity, %; (**c**) Temperature, °C.

The Cu/Al_2_O_3_-SiO_2_ catalyst is assessed in parallel. The catalytic performance of Cu/Al_2_O_3_-SiO_2_ is obviously superior to that of Cu/SiO_2_, as shown in Table 4 and Fig. 4. Most importantly, the stability of Cu/Al_2_O_3_-SiO_2_ catalyst is significantly improved. The conversion of EL on Cu/Al_2_O_3_-SiO_2_ is approximately 100% and the total selectivity of GVL and 2-MTHF is greater than 94% in 1000 h long term test. When the reaction temperature is adjusted between 175 °C and 185 °C, only the relative ratio of GVL and 2-MTHF is sensitively affected.

As shown in Table 4, when the conversion of ethyl levulinate reaches 99.89% at 200 °C, the selectivities of GVL and 2-MTHF are 88.46% and 8.33%, respectively. In contrast to Cu/SiO_2_ catalyst, the reaction temperature is decreased by 30 °C on Cu/Al_2_O_3_-SiO_2_ catalyst under comparable EL conversion and GVL selectivity, which means that its hydrogenation activity is greatly improved through the doping of Al_2_O_3_ into the SiO_2_ support. The Tamman’s temperature of copper is aound 265 °C, which leaves a much larger operation window for the Cu/Al_2_O_3_-SiO_2_ catalyst.

Furthermore, the formation of 1-pentanol, 2-pentanol, and 1,4-pentanediol byproducts is largely suppressed on the Cu/Al_2_O_3_-SiO_2_ catalyst. At 200 °C, the selectivities of 2-pentanol, 1-pentanol, and 1,4-pentanediol are 0.04%, 0.28%, and 0.45%, respectively. It is noteworthy that the selectivity of ethyl valerate slightly increases. Overall, the total selectivity of target GVL and 2-MTHF is improved in contrast to Cu/SiO_2_ catalyst.

To further explore the potentiality of this Cu/Al_2_O_3_-SiO_2_ catalyst, EL conversion and product selectivities at different temperatures are thoroughly investigated and listed in Table 4. At 141 °C, only 79.05% EL is converted and the selectivity of GVL is as high as 95.89%. When the temperature is increased to 151 °C, the EL conversion reaches 98.30%. The GVL selectivity is 94.18% and negligible 0.25% 2-MTHF forms at the same time. This is the lowest temperature ever reported for the hydrogenation of EL to produce GVL selectively on copper based catalysts. Low reaction temperature suppresses the formation of undesired byproducts. The boiling points of EL and GVL are 205.5 °C and 206.6 °C, respectively. High activity catalyst facilitates energy-efficient liquid phase hydrogenation process, eliminating pre-evaporation procedure.

Stepwise increase of the temperature causes drastic selectivity changes. 2-MTHF selectivity keeps increasing at the expense of GVL selectivity. When the temperature reaches as high as 250 °C, 2-MTHF becomes the main product, and the GVL selectivity decreases sharply to 7.96%. The selectivity of 1-pentanol and ethyl valerate reaches 16.78% and 3.52%, respectively, which severely deteriorates the total selectivity of 2-MTHF and GVL. Lee *et al*. reports the 2-MTHF production from LA on Cu/SiO_2_ catalyst^25^. The selectivity of 1-pentanol was above 35% when selectivity of 2-MTHF reaches 64%. So this type of catalyst is not suitable for LA or EL hydrogenation when 2-MTHF is the target product.

### The features of Cu/Al_2_O_3_-SiO_2_ catalyst

**Figure 5:**
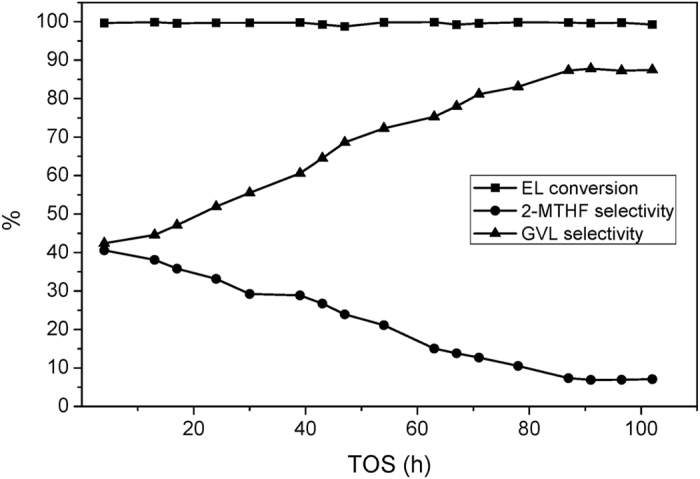
The initial activity of the Cu/Al_2_O_3_-SiO_2_ catalyst on EL hydrogenation.

The initial activity of Cu/SiO_2_ type catalyst is seldom discussed in the previous literature. Figure 5 clearly illustrates the initial activity of this kind of catalyst when it is assessed in fixed bed reactor. After EL feedstock is fed on freshly reduced Cu/Al_2_O_3_-SiO_2_ catalyst at 200 °C and 3.0 MPa, the conversion of EL are nearly 100%. The selectivity of 2-MTHF decreases steadily from 40.57% to 7.35% in the first 90 h, and then stays at ca. 7% thereafter. At the same time, the selectivity of GVL increases steadily from 42.41% to 87.31% in the first 90 h, and then remains at ca. 87% in parallel. So it is advised to adopt low enough initial temperature in the beginning of the reaction.

**Figure 6:**
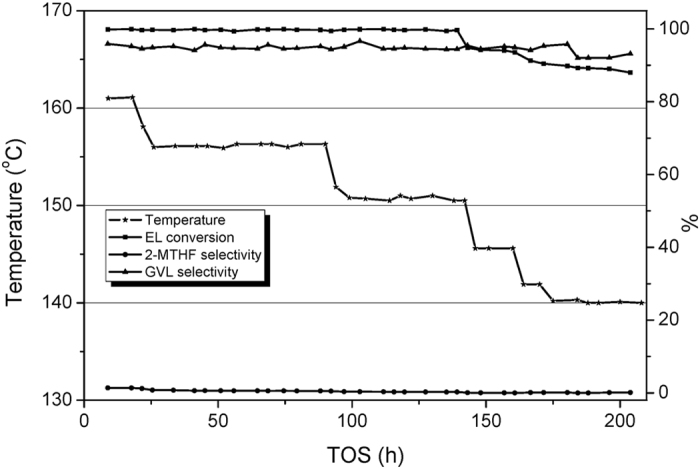
The effect of temperature on the Cu/Al_2_O_3_-SiO_2_ catalyst performance between 140 °C and 162 °C.

**Figure 7:**
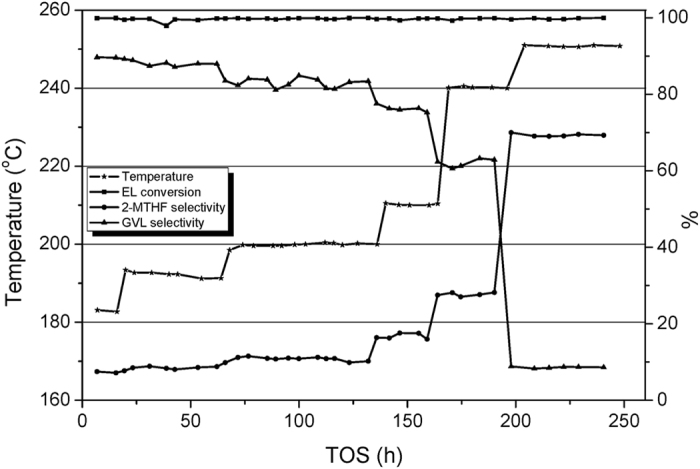
The effect of temperature on the Cu/Al_2_O_3_-SiO_2_ catalyst performance between 170 °C and 250 °C.

Figure 6 clearly illustrates the trends of conversion and selectivity changes when the temperature is adjusted progressively from 161 °C to 140 °C. 151 °C is the lowest temperature that can ensure the entire conversion of EL. Formation of 2-MTHF is negligible between 151 °C and 161 °C. Figure 7 shows the catalytic behaviors when the temperature is increased from 182 °C to 250 °C. Below 240 °C, GVL is the dominant product. Further increasing the temperature to 250 °C, the product distribution reverses. It is manifested that temperature has much greater influence on MTHF selectivity in 240–250 °C temperature range than that under lower temperature.

**Figure 8:**
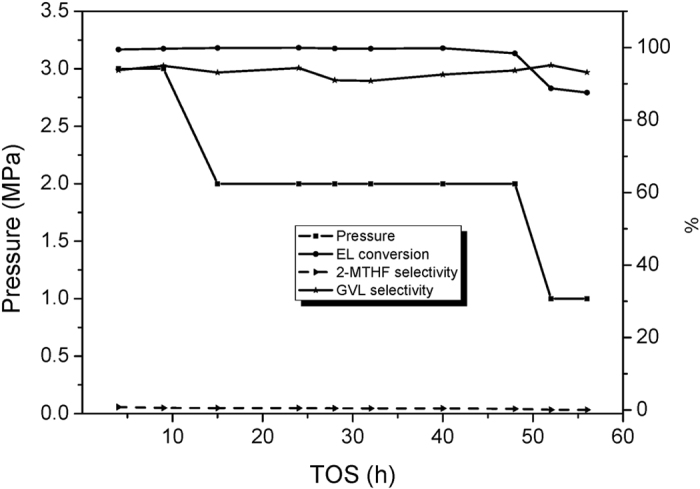
The effect of pressure on the Cu/Al_2_O_3_-SiO_2_ catalyst performance.

Pressure also has notable influence on the performance of Cu/Al_2_O_3_-SiO_2_ catalyst, as shown in Fig. 8. 3.0 MPa, 2.0 MPa, and 1.0 MPa conditions are undertaken to study the pressure effect. The above reported data are obtained under 3.0 MPa pressure, which means this pressure condition can assure the long term run of the catalyst. When the pressure is decreased to 2.0 MPa, the conversion of EL shows a declining trend in less than 40 h. Under 1.0 MPa, the conversion of EL decreases sharply to below 90%.

### Reaction pathways and the effect of Al_2_O_3_ doping

**Figure 9:**
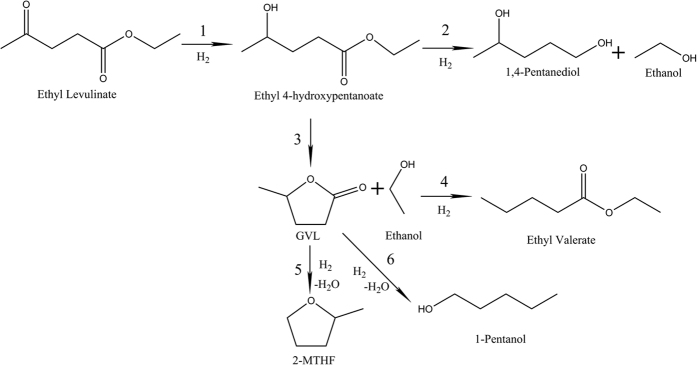
The proposed reaction pathways of ethyl levulinate hydrogenation.

Figure 9 proposes the possible reaction pathways from EL to all the identified products. The formation mechanism of GVL and 2-MTHF in the hydrogenation of EL is well established^16^. In the first place, EL is selectively saturated to form ethyl 4-hydroxypentanoate (reaction 1). The cyclization reaction leads to the formation of GVL and ethanol (reaction 3). The further hydrogenation of GVL results in the formation of 2-MTHF under harsher conditions (reaction 5). The sequential reaction of EL to 2-MTHF via GVL is the main reaction route.

In order to minimize the production of undesired byproducts, the formation mechanism of main by-products also deserves further examination (Fig. 9). The relative selectivity of 2-MTHF, GVL, 2-pentanol, 1-pentanol, 1,4-pentanediol, and ethyl valerate at different temperatures gives important implication on the reaction pathways of EL hydrogenation. Unlike 2-pentanol, 1-pentanol, and ethyl valerate, the selectivity of 1,4-pentanediol doesn’t change largely when the temperature is above 160 °C. It is indicated that 1,4-pentanediol (reaction 2) may be hydrogenated from ethyl 4-hydroxypentanoate intermediate. The support acidity directly drives the formation of ethyl valerate in bifunctional catalyst^29^. As proposed by Dumesic *et al*., protonation of GVL is the initial step of the acid-catalyzed ring-opening of GVL to pentenoic acid^30^. Therefore, the reaction 4 is somewhat strengthened on Cu/Al_2_O_3_-SiO_2_ catalyst. The sequential hydrogenation of GVL via pentanal intermediate produces 1-pentanol (reaction 6), which competes with the route from GVL to 2-MTHF. This reaction is pronounced especially under high temperature. 2-pentanol might be the hydrodeoxygenation product of 1,4-pentanediol.

The doping of Al_2_O_3_ in the SiO_2_ support plays crucial role in the higher activity, selectivity, and longevity in EL hydrogenation reaction. The doping of Al_2_O_3_ decreases the particle sizes of CuO clusters in the co-precipitation process. Smaller Cu particles after reduction and hydrogen spillover from Cu to Lewis acidic sites may account for the much improved activity of the Cu/Al_2_O_3_-SiO_2_ catalyst. The CuO particles distribute around 20–60 nm in the spent and re-calcined Cu/Al_2_O_3_-SiO_2_ catalyst (Figure S3). Sintering of Cu particles is almost negligible during long-term operation, which also accounts for the good stability of this Cu/Al_2_O_3_-SiO_2_ catalyst. Sintering inhibition by anchoring of Cu particles on the Lewis acidic sites and much lower reaction temperature may help to the greatly improved stability of the Cu/Al_2_O_3_-SiO_2_ catalyst. The stronger Lewis acidic sites also accelerates the dehydration reaction to form GVL main product, suppressing the formation of 1,4-pentanediol and 2-pentanol.

For the production of γ-valerolactone from ethyl levulinate, Al_2_O_3_ doped Cu/SiO_2_ catalyst is developed by co-precipitation method. Compared to Cu/SiO_2_ catalyst, this catalyst shows largely enhanced activity, high γ-valerolactone selectivity, and long period stability. The effect of support acidity on catalytic performance is manifested. The introduction of alumina in SiO_2_ support improved the activity greatly. Ethyl levulinate is totally converted to γ-valerolactone at as low as 151 °C. Low reaction temperature suppresses the formation of undesired byproducts. Cu/Al_2_O_3_-SiO_2_ catalyst fully satisfies the stability requirement for continuous fixed bed application. This work proves the feasibility for continuous fixed bed hydrogenation of ethyl levulinate to produce γ-valerolactone based on this environmentally friendly copper catalyst.

## Methods

### Catalyst preparation and characterization

The Cu/SiO_2_ catalyst was prepared by the co-precipitation method in which 0.20 mol/L aqueous solution of Cu(NO_3_)_2_.3H_2_O and proper amount of silica sol were taken and precipitated using 0.2 mol/L aqueous sodium carbonate at ambient temperature. The precipitate was aged further for 8 h at 60 °C. Then the mixture was separated by filtration and washed with deionized water to remove the traces of sodium. The solid thus obtained was dried in static oven at 110 °C for 24 h and calcined at 400 °C for 4 h. The weight percentage of copper in the Cu/SiO_2_ catalyst was 20.5%.

In parallel, alumina doped Cu/SiO_2_ catalyst was prepared by the co-precipitation method in which 0.20 mol/L aqueous solution of Cu(NO_3_)_2_.3H_2_O, silica sol, and proper amount of alumina sol were taken and precipitated using 0.2 mol/L aqueous sodium carbonate at ambient temperature. The precipitate was aged further for 8 h at 60 °C. Then the mixture was separated by filtration and washed with deionized water to remove the traces of sodium. The solid thus obtained was dried in static oven at 110 °C for 24 h and calcined at 400 °C for 4 h. The SiO_2_ to Al_2_O_3_ molar ratio was 28.5 in the final alumina doped Cu/SiO_2_ catalyst. The weight percentage of copper in the Cu/Al_2_O_3_-SiO_2_ catalyst was 20.3%.

X-ray powder diffraction (XRD) patterns of series CuO/SiO_2_ catalyst were recorded in the range of 5~80° on Bruker D8 diffractometer using nickel-filtered Cu Kα radiation with a scanning step 0.02°, voltage 40 kV, and current 100 mA. High resolution Transmission Electron Microscopy (TEM) was recorded on a FEI TECNAI-20 instrument with accelerating voltage of 200 kV. TEM specimen was prepared by dispersing the powder in alcohol by ultrasonic treatment and dropping onto a holey carbon film supported on a copper grid, and then dried in air. The pyridine adsorption infrared spectroscopy (Py-IR) of the two catalysts was collected on a Nicolet 5700 infrared instrument with homemade vacuum adsorption system. Samples were activated at 400 °C and then vacuumed to less than 0.1 Pa in the sealing system. Adsorption occurred at room temperature in a saturated atmosphere of pyridine for 30 min, followed by degassing to less than 0.1 Pa. After pyridine adsorption, the sample was heated to 200 °C, 250 °C, 300 °C, and 350 °C stepwise. Then, Py-IR spectroscopy was collected.

XRD patterns of H_2_ reduced Cu/SiO_2_ and Cu/Al_2_O_3_-SiO_2_ catalysts were collected, and the Cu particle sizes were calculated by scherrer equation, respectively. Furthermore, the Cu dispersion was determined by dissociative N_2_O chemisorptions^31^. The copper dispersion of the catalysts was determined by dissociative N_2_O chemisorptions on surface copper atoms. Briefly, 100 mg of calcined catalysts were reduced in 5% H_2_–95% Ar at 623 K for 4 h and cooled to 333 K. Then pure N_2_O was introduced at a rate of 30 cm^3^ min^−1^ for 1 h, ensuring that surface Cu atoms were completely oxidized according to the reaction 2Cu(s) + N_2_O → Cu_2_O(s) + N_2_. The quantity of irreversibly chemisorbed O_2_ atoms was measured by a hydrogen pulse chromatographic technique on a Micromeritics Autochem II 2920 equipped with a TCD. Hydrogen pulse reduction of surface Cu_2_O to metallic copper was conducted at 573 K to ensure that the chemisorbed oxygen on the copper surface immediately reacted with pure hydrogen gas introduced from the pulse loop to form water, and the formed water was soon removed by a 13X molecule sieve dehydration tube in the reaction outlet. Hydrogen pulse-dosing was repeated until the pulse area no longer changed. The consumed amount of hydrogen was the value obtained by subtracting the small area of the first few pulses from the area of the other pulses. Copper dispersion (D_Cu_) was calculated by dividing the amount of chemisorptions sites into total supported copper atoms. Cu surface area (S_Cu_) and mean Cu particle size (d_Cu_) for the Cu/SiO_2_ and Cu/Al_2_O_3_-SiO_2_ catalysts were calculated accordingly.

### Catalyst test and analysis

EL (purity >99.5%) was purchased from local company and used without further treatment. The performance of the catalysts was assessed on a fixed bed reactor (i.d. 12 mm, length 500 mm), into which 5 g of catalyst was loaded. Prior to the reaction, the catalyst was reduced at 400 °C in H_2_ flow. EL and ethanol solvent (EL/ethanol volume ratio was 1/1) were fed into the reactor using an injection pump, and the WHSV flow rate of EL was 0.6 h^−1^. The reaction temperature was adjusted between 141 °C and 251 °C. The reaction pressure was maintained at 1.0–3.0 MPa. The molar ratio of hydrogen to EL was set as 50 (mol/mol). The hydrogenation products were analyzed using gas chromatography (Agilent 7890) equipped with a flame ionization detector and a capillary column (DB-200). The main products and byproducts were identified by GC-MS method on Agilent 5975C inert XL EI/CI MSD. EL conversion, GVL selectivity, and 2-MTHF selectivity were calculated using the following equations:


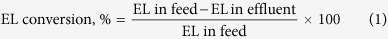



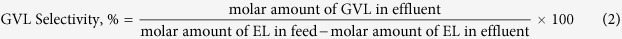



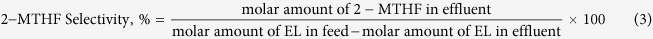


The selectivity of other byproducts, such as 2-pentanol, 1-pentanol, 1,4-pentanediol, and ethyl valerate, were calculated using the identical equations.

## Additional Information

**How to cite this article**: Zheng, J. *et al*. Continuous hydrogenation of ethyl levulinate to γ-valerolactone and 2-methyl tetrahydrofuran over alumina doped Cu/SiO_2_ catalyst: the potential of commercialization. *Sci. Rep.* **6**, 28898; doi: 10.1038/srep28898 (2016).

## Supplementary Material

Supplementary Information

## Figures and Tables

**Table 1:** Cu dispersion data as determined by dissociative N_2_O chemisorptions on surface copper atoms.

**Catalyst**	**D**_**Cu**_ **(%)**	**S**_**Cu**_ **(m**^**2**^**g**^**−1**^**Cu)**	**d**_**Cu**_ **(nm)**	**d**_**Cu**_ **(nm)**&
Cu/SiO_2_	4.00	5.04	27.01	22.40
Cu/Al_2_O_3_-SiO_2_	4.69	5.91	23.02	19.10

^&^Cu particle sized calculated from XRD patterns of H_2_ reduced catalysts by Scherrer equation.

**Table 2:** Py-IR characterization of Cu/SiO_2_ and Cu/Al_2_O_3_-SiO_2_ catalysts.

**Catalyst**	**Acid**	**Acidity (×10**^**−2**^**, μmol/g)**			
		**200 °C**	**250 °C**	**300 °C**	**350 °C**
Cu/SiO_2_	Brönsted	0	0	0	0
	Lewis	1.50	1.00	0	0
Cu/Al_2_O_3_-SiO_2_	Brönsted	0	0	0	0
	Lewis	2.00	1.37	1.00	0.0

**Table 3:** Products distribution in the ethyl levulinate hydrogenation effluents on Cu/SiO_2_ catalyst under different temperatures.

**T (°C)**	**Conversion (%)**	**2-MTHF (%)**	**2-pentanol (%)**	**1-pentanol (%)**	**GVL (%)**	**PND (%)**&	EV (%)*	**UI (%)**#
151	83.67	0	0	0	97.21	0	0	2.79
160	88.43	0.17	0	0	96.59	0.15	0	3.09
172	95.02	1.68	0.35	0.14	93.81	1.30	0.05	2.67
200	98.76	3.05	2.05	0.89	90.23	1.67	0.08	2.03
230	98.95	7.18	2.95	1.24	85.06	2.04	0.10	1.43

^&^1,4-Pentanediol.

^*^Ethyl Valerate.

^#^Unidentified.

**Table 4:** Product distribution in the ethyl levulinate hydrogenation effluents on Cu/Al_2_O_3_-SiO_2_ catalyst under different temperatures.

**T (°C)**	**Conversion (%)**	**2-MTHF (%)**	**2-pentanol (%)**	**1-pentanol (%)**	**GVL (%)**	**PND (%)**&	EV (%)*	**UI (%)**#
141	79.05	0	0	0	95.89	0	0	4.11
151	98.30	0.25	0	0	94.18	0.21	0	5.36
160	99.83	1.40	0	0.05	95.82	0.47	0.11	2.15
172	99.69	1.91	0	0.07	94.62	0.30	0.14	2.96
200	99.89	8.33	0.04	0.28	88.46	0.45	0.28	2.44
210	99.84	16.38	0.09	1.57	77.58	0.43	0.54	3.41
240	99.99	27.50	0.17	3.56	62.35	0.45	0.63	5.34
250	99.99	65.18	0.54	16.78	7.96	0.51	3.52	5.51

^&^1,4-Pentanediol.

^*^Ethyl Valerate.

^#^Unidentified.
