# Supplementary material for: Continuous hydrogenation of ethyl levulinate to γ-valerolactone and 2-methyl tetrahydrofuran over alumina doped Cu/SiO2 catalyst: the potential of commercialization
Source: Sci Rep. 2016 Jul 5;6:28898. doi: 10.1038/srep28898 (PMC4932554; doi:10.1038/srep28898)
Supplement: Supplementary Information [file srep28898-s1.pdf]

Supporting information for

Continuous hydrogenation of ethyl levulinate to γ-valerolactone and 2-methyl tetrahydrofuran over alumina doped Cu/SiO_2 catalyst: the potential of commercialization

Junlin Zheng[2]†, Junhua Zhu[1,2]†, Xuan Xu[2], Wanmin Wang[2], Jiwen Li[2], Yan Zhao[2], Kangjian Tang[1,2], Qi Song[2], Xiaolan Qi[2], Dejin Kong[2], Yi Tang[1]

1 Department of Chemistry, Fudan University, Shanghai, 200233, China

2 Shanghai Research Institute of Petrochemical Technology SINOPEC, Shanghai 201208, China.

† co-first author with equal contribution

XRD patterns of H_2 reduced Cu/SiO_2 and Cu/Al_2O_3-SiO_2 catalysts were collected (as shown in Figure 1S), and the Cu particle sizes were calculated by scherrer equation, respectively. After H_2 reduction, the copper particle sizes of Cu/SiO_2 and Cu/Al_2O_3-SiO_2 are 22.40 nm and 19.10 nm, respectively.

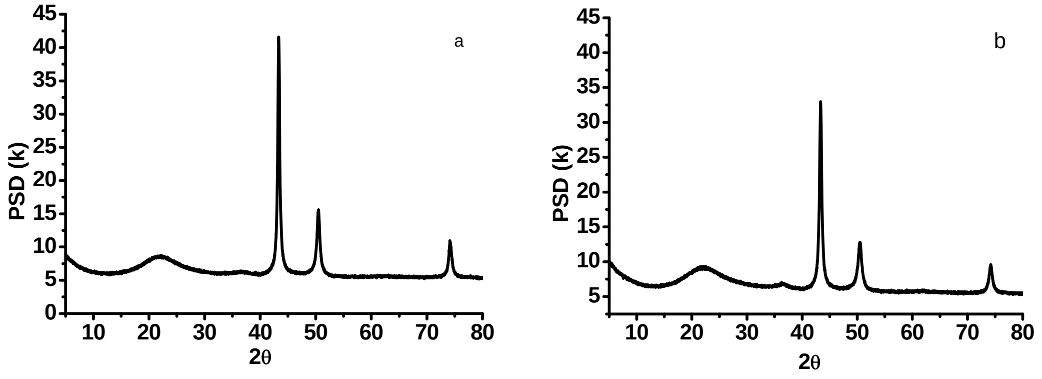

Figure S1. XRD patterns of H_2 reduced Cu/SiO_2 (a) and Cu/Al_2O_3-SiO_2 (b) catalysts.

Figure S2 displays the Py-IR spectroscopy of the Cu/SiO_2 (a) and Cu/Al_2O_3-SiO_2 (b) catalysts desorbed at 200 °C, 250 °C, 300 °C, and 350 °C stepwise. Normally, the peak at 1450 cm^{-1} and 1540 cm^{-1} are ascribed to Lewis acidic sites and Brönsted acidic sites, respectively. The Cu/SiO_2 catalyst only has very weak Lewis acidic sites, as indicated by the desorption signal at 200 °C and 250 °C. Whereas, the Cu/Al_2O_3-SiO_2 catalyst possesses stronger Lewis acidic sites, as indicated by the stronger desorption signals at 200 °C and 250 °C, as well as the additional desorption signals at 300 °C.

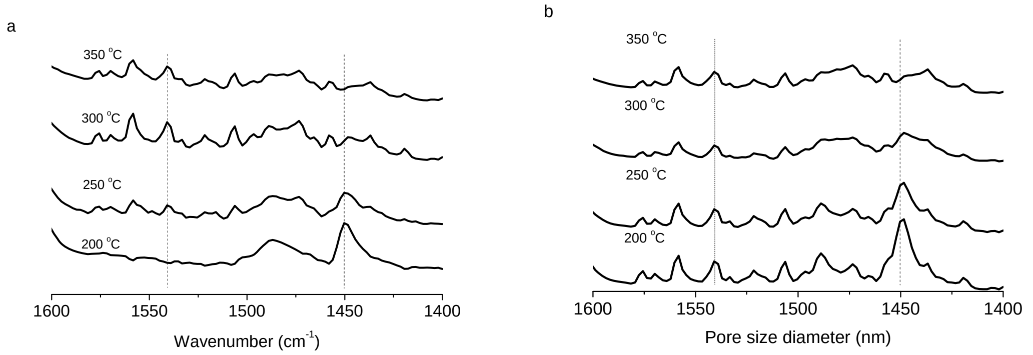

Figure S2. Py-IR characterization of CuO/SiO_2 (a) and CuO/Al_2O_3-SiO_2 (b) catalysts.

Figure S3a shows the TEM micrograph of spent Cu/Al_2O_3-SiO_2 catalyst after 1000 h test. The copper particles are not clearly displayed. The spent catalyst was re-calcined at 500 ^oC for 2 h in the air, and the morphology of CuO particles in TEM micrograph is manifested in Figure S3b. The CuO particles distribute around 20-60 nm. Sintering of Cu particles was not remarkable during long-term operation, which also accounted for the good stability of this Cu/Al_2O_3-SiO_2 catalyst.

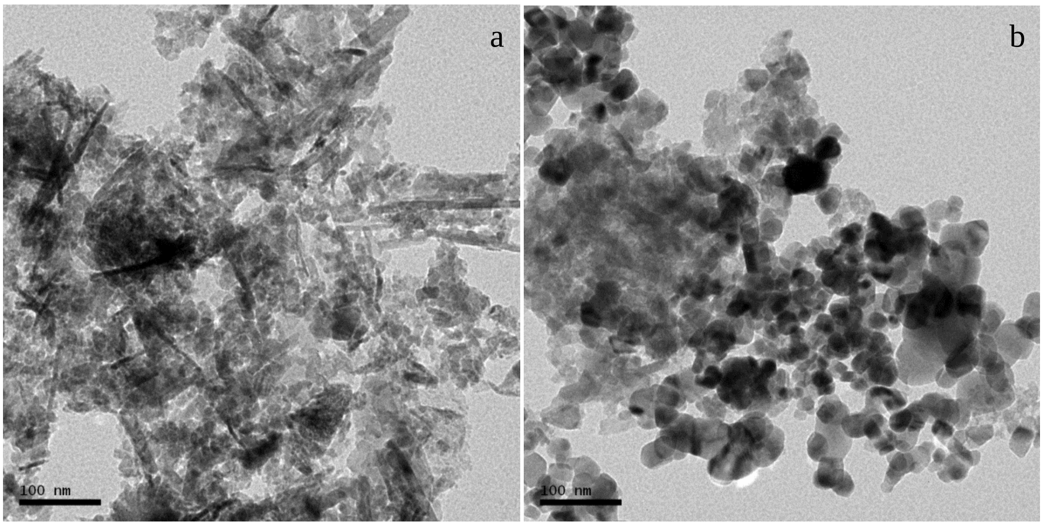

Figure S3. TEM micrographs of spent Cu/Al_2O_3-SiO_2 catalyst: (a) after 1000 h test; (b) re-calcined at 500 ^oC for 2 h in air.
